# Real-world cardiovascular risks of ibrutinib in chronic lymphocytic leukemia: a retrospective study

**DOI:** 10.3389/fonc.2025.1624761

**Published:** 2025-08-26

**Authors:** Jelena Ivanovic, Vladimir Otasevic, Ksenija Markovic, Vojin Vukovic, Tamara Bibic, Kristina Tomic Vujovic, Sofija Kozarac, Jelena Vladicic Masic, Natasa Milic, Jovan Kulic, Darko Antic

**Affiliations:** ^1^ Clinic for Hematology, University Clinical Center of Serbia, Belgrade, Serbia; ^2^ Institute for Medical Statistics and Informatics, Faculty of Medicine, University of Belgrade, Belgrade, Serbia; ^3^ Faculty of Medicine, University of Belgrade, Belgrade, Serbia; ^4^ Department of Internal Medicine, Faculty of Medicine Foca, University of East Sarajevo, Foca, Bosnia and Herzegovina; ^5^ Center for Biomedical Sciences, Faculty of Medicine Foca, University of East Sarajevo, Foca, Bosnia and Herzegovina

**Keywords:** chronic lymphocytic leukemia, cardiovascular risk, drug toxicity, ibrutinib, BTKi, cardiotoxicity

## Abstract

**Introduction:**

Ibrutinib has made significant contributions to the treatment of chronic lymphocytic leukemia (CLL) with recognized cardiovascular toxicities in some patients. This study aimed to assess the incidence of cardiotoxicity in CLL patients treated with ibrutinib and identify associated risk factors.

**Methods:**

This retrospective cohort study analyzed 79 CLL patients treated with ibrutinib at the University Clinical Center of Serbia. Patient characteristics, treatment outcomes, and cardiovascular events were analyzed to determine the incidence of cardiotoxicity and its potential predictors.

**Results:**

The median age at diagnosis was 58 years, with 63.3% male patients. Pre-existing cardiovascular conditions were present in 55.7% of patients. Cardiotoxicity occurred in 29.1% of patients, with atrial fibrilation developing in 10.1% patients (37.5% grade 3), leading to therapy discontinuation in 62.5% of those affected. Also, we diagnosed hypertension in 15.2%, heart failure in 7.6%, and myocardial infarction in 2.5% of patients. Furthermore, one case (1.3%) of sudden cardiac death was recorded. The administration of ibrutinib was ceased in 9 patients due to cardiotoxic effects. Patients with prior cardiovascular disease had a threefold increased risk of developing cardiotoxicity (HR=2.850; p=0.031). A history of hypertension was significantly associated withthe worsening of hypertension during ibrutinib therapy (HR=7.935; p=0.009). No significant associations were found between cardiotoxicity and other factors such as age, sex, number of prior treatment lines, clinical stage, or cytogenetic abnormalities.

**Discussion:**

This study underscores the importance of cardiovascular monitoring in CLL patients undergoing ibrutinib therapy, particularly those with pre-existing cardiovascular conditions. These findings highlight the need for individualized treatment planning and close follow-up to mitigate the risk of cardiotoxicity and optimize patient outcomes.

## Introduction

Among the various forms of leukemia, chronic lymphocytic leukemia (CLL) stands out as the most common in Western adult populations, comprising around 1.2% of all malignancies (1, 2). Its clinical significance continues to drive advances in both research and treatment. The disease primarily impacts older adults. Recent advances have transformed the management of CLL and outcomes in patients with this disease. The management of CLL has evolved significantly with the advent of targeted therapies, particularly Bruton’s tyrosine kinase inhibitors (BTKi), that have become the cornerstone of modern treatment for CLL, essentially replacing traditional chemoimmunotherapy regimens. Treatment selection is guided by genetic and clinical factors, including TP53 mutation status, 17p deletion (del17p), immunoglobulin heavy-chain variable region gene (IGHV) mutational status, comorbidities, patient fitness, and individual preferences (3, 4).

Ibrutinib, the first approved BTKi, revolutionized CLL treatment by inducing durable remissions, even in high-risk patients. It has shown high efficacy and favorable survival outcomes, both in treatment-naïve and relapsed/refractory patients (5–9). It works by inhibiting BTK, a key enzyme in B-cell receptor signaling, thereby preventing malignant cell proliferation and survival. However, despite its therapeutic benefits, ibrutinib is associated with a range of adverse events, including cardiovascular toxicities, which may impact its overall safety profile and clinical outcomes (10).

Cardiovascular diseases represent the second most prevalent cause of morbidity and mortality among cancer patients (11). While the advent of targeted therapies has markedly enhanced treatment outcomes in chronic lymphocytic leukemia (CLL), the cardiotoxic effects associated with these therapies remain a significant concern. Such adverse cardiovascular events can still manifest with targeted treatments and, in certain instances, may necessitate the interruption or cessation of treatment, highlighting the imperative for diligent cardiac monitoring throughout the therapeutic process.

The aim of this study is to assess the incidence of cardiotoxicity in CLL patients treated with ibrutinib and identify potential risk factors for cardiovascular adverse events, with a particular focus on the role of pre-existing cardiovascular conditions. This analysis is based on real-world data, aiming to better understand the development of cardiotoxicity during ibrutinib therapy. Given the growing use of ibrutinib in CLL management, understanding the risk of cardiotoxicity is critical for optimizing treatment strategies and improving patient outcomes.

## Materials and methods

This retrospective cohort study includes 79 consecutive patients diagnosed and treated with ibrutinib in the University Clinical Center of Serbia. Ibrutinib was administered as per approved dosing regimens for CLL. Demographic, clinical, laboratory, and cytogenetic data were extracted from electronic medical records. Cytogenetic abnormalities were assessed using fluorescence *in situ* hybridization (FISH), and conventional karyotyping was performed where available at the time of first treatment. A complex karyotype was defined as the presence of three or more chromosomal abnormalities detected in a single clone using conventional cytogenetic analysis. Cardiovascular comorbidities and adverse events were defined based on patient history and standard diagnostic criteria. Cardiotoxicity was graded according to Common Terminology Criteria for Adverse Events 5.0 (CTCAE). Categorical variables are displayed as counts with percentages, and numerical variables are presented as means with standard deviations or medians with 25th-75th percentiles (according to data distribution). Normality of distribution was assessed using the Kolmogorov–Smirnov test. Differences between patients with cardiotoxicity and those without cardiotoxicity were assessed using the Student’s t test or Mann-Whitney test for numerical variables and the chi-square test for categorical variables. Univariate and multivariate Cox regression analyses were performed to identify significant predictors of cardiotoxicity in patients with CLL receiving Ibrutinib. Significant variables from the univariate logistic regression analysis were fitted into the multivariate analysis if p<0.1. The results are presented as hazard ratios (HRs) with corresponding 95% confidence intervals (CIs). Overall survival and cumulative incidence were assessed using Kaplan–Meier survival analyses. In the case of an event, the time of the event was recorded. For censored observations without an event, the time of the last follow-up was used. Statistical significance was set at p < 0.05. Statistical analysis was performed using IBM SPSS statistical software (SPSS for Windows, release 25.0, SPSS, Chicago, IL, USA).

## Results

The median age at diagnosis was 58 years (range: 26–79), with 50 patients (63.3%) being male. The median age of the patients at the time of Ibrutinib treatment was 65 years (range: 27–89). According to the Rai classification, clinical stage (CS) at the time of Ibrutinib initiation was distributed as follows: stage 0 in 2.5%, stage 1 in 17.7%, stage 2 in 38%, stage 3 in 10.1%, and stage 4 in 31.6% of patients. The median pretreatment hemoglobin level was 126 g/L (105–136 g/L), leukocyte count was 70.8 × 10^9^/L (12.9-172.3 × 10^9^/L), and platelet count was 127 × 10^9^/L (94-180× 10^9^/L). FISH analysis revealed cytogenetic abnormalities in a significant proportion of patients: 59.2% had a 17p deletion, 53.3% had a 13q deletion, 30.3% had an 11q deletion, while 6.6% had trisomy 12. FISH data were unavailable for three patients, and additionally trisomy 12 status was missing in four patients. Conventional cytogenetic testing was performed in 59 patients (74.7%), of whom 37.3% had a complex karyotype. Institutional protocol recommended FISH and cytogenetic analysis before the start of any line of therapy. The median interval between diagnosis and the initiation of Ibrutinib therapy was 55 months (range: 23–88 months). Ibrutinib was administered as first-line therapy in 20 patients (25.3%). Among the remaining patients, it was introduced after disease relapse: 35.4% following one prior line of therapy, 21.5% after two lines, 12.7% after three lines, and 5.1% after four lines. None of the patients had prior exposure to other BTK inhibitors.

A personal history of cardiovascular disease, such as hypertension, angina pectoris, myocardial infarction, or AF, was present in 44 patients (55.7%) before starting ibrutinib. Hypertension was the most prevalent (n=42, 53.2%), while myocardial infarction was present in 6 patients (7.6%), AF in 4 patients (5.1%) and angina pectoris in 2 patients (2.5%).

The median duration of ibrutinib therapy was 22 months (25^th^ to 75^th^ percentile: 10 to 31 months). At the time of analysis, 44 patients (55.7%) remained on treatment, while therapy was discontinued in 44.3% of cases. The primary reason for discontinuation was adverse events, accounting for 34.3% of all treatment cessations. Among these, 8.3% were due to hematological toxicity, 25% due to infectious complications, and the largest proportion, 66.7%, was attributed to cardiotoxicity. The administration of Ibrutinib was ceased in 9 patients due to cardiotoxic effects. Infectious complications leading to treatment discontinuation were most commonly respiratory tract infections, followed by COVID-19, sepsis, and a skin and soft tissue infection.

**Figure 1:**
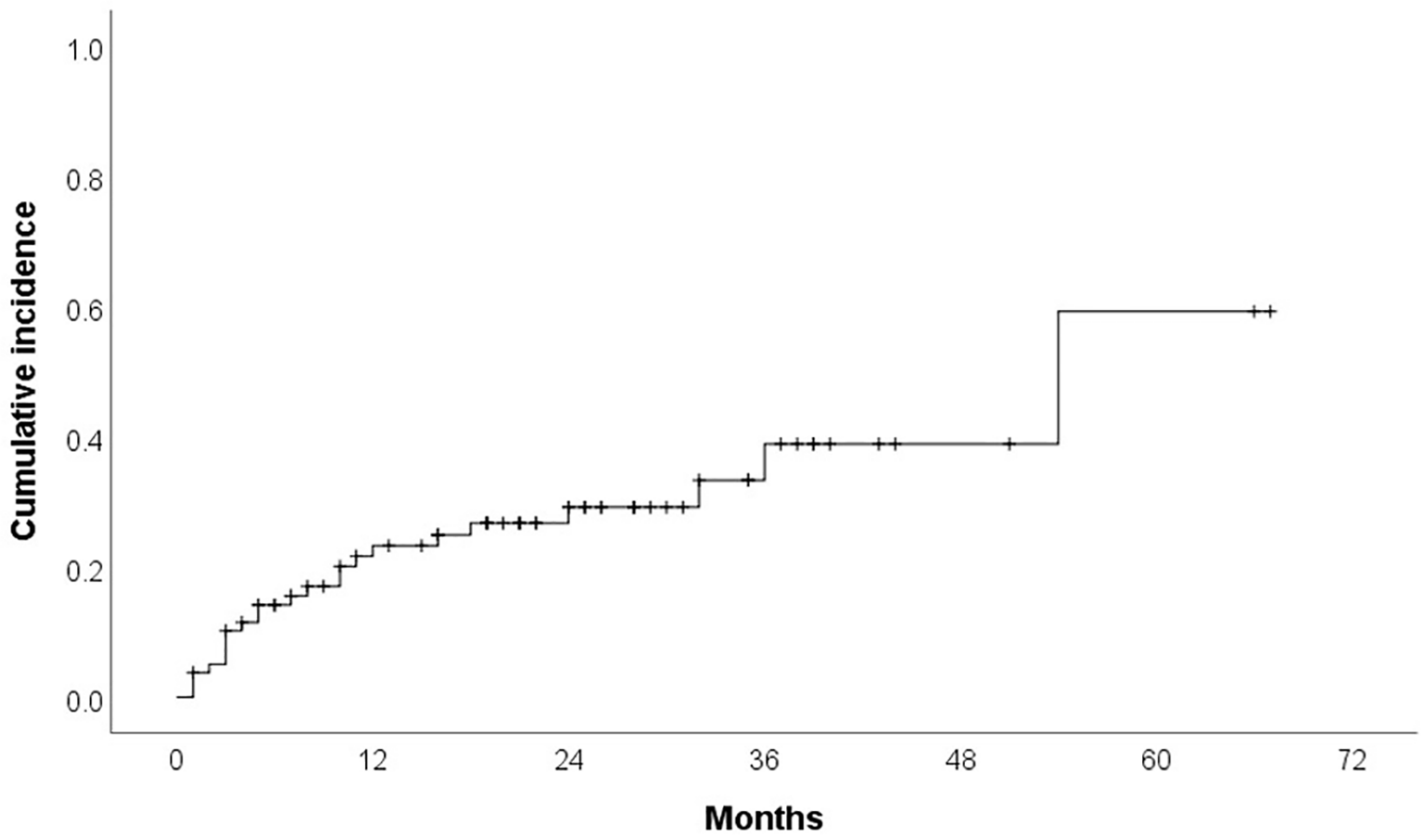
Cumulative incidence Kaplan Meier curve for cardiotoxicity.

**Figure 2:**
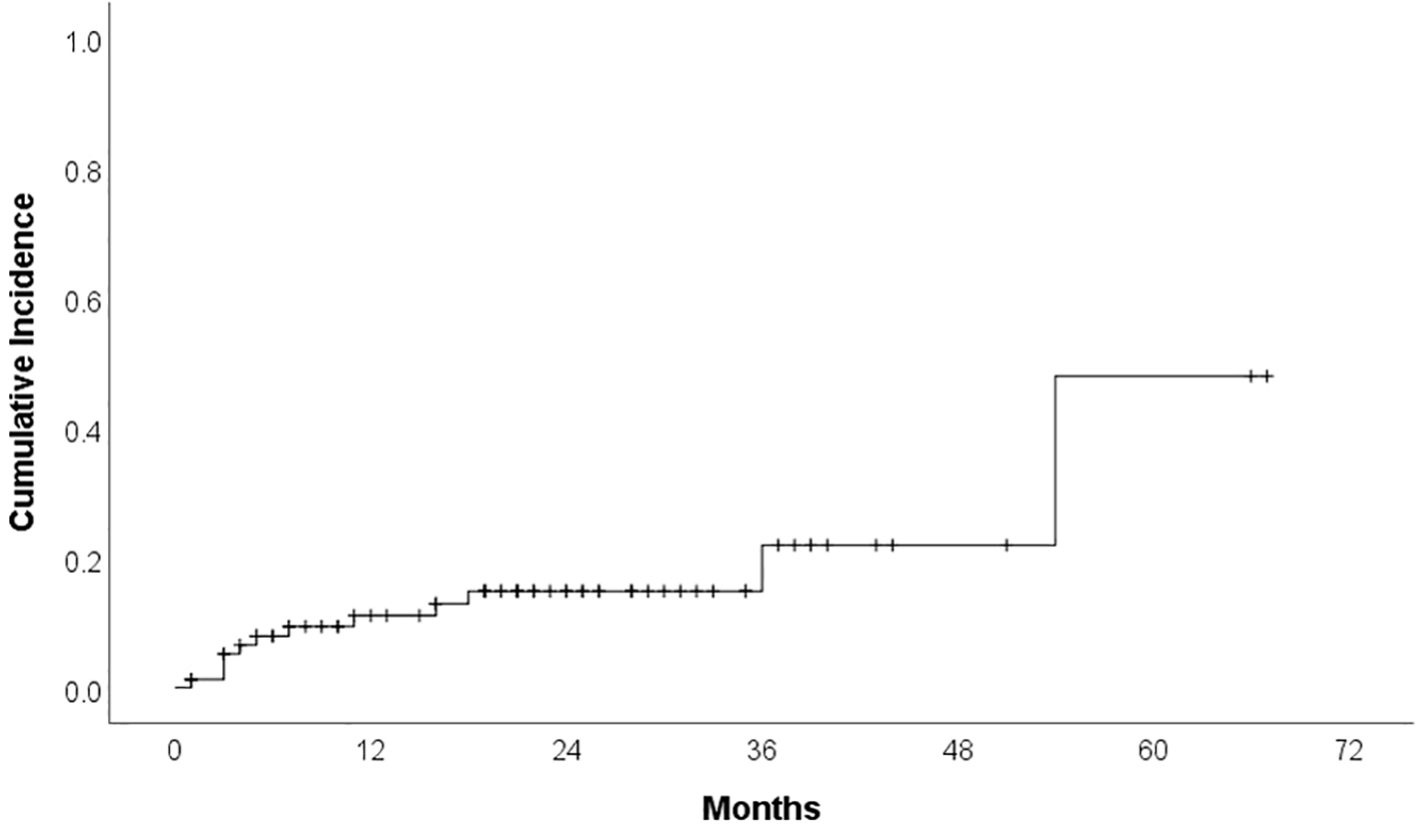
Cumulative incidence Kaplan Meier curve for hypertensive toxicity.

Cardiotoxicity occurred in 23 patients (29.1%) during treatment. Median time to cardiotoxicity development was 7 months. Hypertension was reported in 12 patients (15.2%) (16.7% grade 1, 25% grade 2, 58.3% grade 3), with ibrutinib discontinued in 16.7% of these cases (median time to development: 6 months). Cumulative incidence curves for overall cardiotoxicity and hypertensive toxicity are presented in **Figures 1** and **2**. Atrial fibrillation (AF) developed in 8 patients (10.1%) (25% grade 1, 37.5% grade 2, 37.5% grade 3), leading to therapy discontinuation in 62.5% of those affected (median time to development: 11 months). We do not have other type of arrhytmias confirmed in our group. Heart failure was documented in 6 patients (7.6%) (33.3% grade 3, 66.7% grade 4), all of whom discontinued therapy (median time to development: 10 months). Acute myocardial infarction occurred in 2 patients (2.5%), leading to treatment cessation in both. Furthermore, one case of sudden cardiac death was recorded.

Demographic and clinical factors according to overall cardiotoxicity and development of hypertension are presented in **Table 1**. Of the 12 patients who experienced hypertension as an adverse event, 11 had a documented history of hypertension before starting treatment, while only one patient developed it without a prior diagnosis. Of the 23 patients who experienced cardiotoxicity, 17 had a documented history of cardiovascular diseases before starting treatment. These results provide important insights into the risk factors associated with the development of cardiotoxicity following the administration of ibrutinib. Individuals with a history of hypertension demonstrated a significant association with the manifestation of hypertension as a form of cardiotoxicity, through the exacerbation of their existing condition (p=0.004). Furthermore, the presence of cardiovascular disease was significantly associated with overall cardiotoxicity (p=0.037). No statistically significant associations were found between cardiotoxicity and patient sex (p = 0.189); age at diagnosis and at the time of Ibrutinib treatment (p = 0.608 and p=0.270); number of prior treatment lines (p = 0.920); clinical stage at diagnosis (p = 0.906); leukocyte count (p = 0.503); hemoglobin level (p = 0.961); platelet count (p = 0.710); and cytogenetic abnormalities including those identified through conventional cytogenetic and FISH (p > 0.05) (**Table 1**). No statistically significant associations were found between hypertensive toxicity and all abovementioned characteristics (**Table 1**).

**Table 1:** Demographic and clinical characteristics according to overall cardiotoxicity and hypertensive toxicity.

Variable	Overall cardiotoxicity		Hypertensive toxicity	
	No	Yes	No	Yes
Sex, male	38 (67.9%)	12 (52.2%)	45 (67.2%)	5 (41.7%)
Age at diagnosis, yrs	58 (53-64)	61 (52-64)	58 (53-64)	55 (47-64)
Age at IBR treatment, yrs	63 (58-69)	66 (58-71)	65 (59-70)	59 (52-67)
n˚ prior lines, first line	14 (25.0%)	6 (26.1%)	17 (25.4%)	3 (25.0%)
RAI clinical stage, >2	24 (42.8%)	9 (39.1%)	29 (43.3%)	4 (33.4%)
Leukocyte count, ×10^9^/L	48.0(11.3-171.9)	89.3(19.5-175.0)	58.0(11.4-172.3)	87.1(20.1-172.5)
Hemoglobin level, g/L	121 (103-138)	127 (105-136)	123 (105-137)	128 (93-133)
Platelet count, ×10^9^/L	122 (93-172)	135 (97-183)	124 (94-173)	133 (92-194)
del17p	31 (57.4%)	14 (63.6%)	39 (60.0%)	6 (54.5%)
del13	30 (56.6%)	10 (45.5%)	35 (54.7%)	5 (45.5%)
del11q	17 (31.5%)	6 (27.3%)	17 (26.2%)	6 (54.5%)
trisomy 12	3 (5.6%)	2 (9.1%)	5 (7.7%)	0 (0%)
Complex karyotype	18 (42.9%)	4 (23.5%)	19 (37.3%)	3 (37.5%)
Previous CVD	27 (48.2%)	17 (73.9%)*	33 (49.3%)	11 (91.7%)*
Previous hypertension	26 (46.4%)	16 (69.6%)	31 (46.3%)	11 (91.7%)*

IBR, Ibrutinib; CVD, cardiovascular disease.

*p<0.05.

Univariate and multivariate Cox regression analysis demonstrated that the presence of cardiovascular disease was a significant predictor of overall cardiotoxicity as adverse reaction (p = 0.031; HR = 2.850; 95% CI: 1.097–7.169) (**Table 2**). It was shown that patients with a history of cardiovascular disease had almost a 3-fold higher likelihood of developing cardiotoxicity compared to those without such prior conditions. Similarly, hypertension emerged as an independent and statistically significant predictor of hypertensive toxicity. Univariate and multivariate analysis indicated that patients with previously diagnosed hypertension were at significantly greater risk of developing this adverse reaction (p = 0.009; HR = 7.935; 95% CI: 1.677–37.557). The risk of hypertension development was almost than eight times higher in patients with hypertension compared to those without (**Table 3**) and it was more pronounced in women (p = 0.031; HR = 3.463; 95% CI: 1.117–10.730).

**Table 2:** Univariate and multivariate Cox regression analyses of risk factors for overall cardiotoxicity.

Risk factor	P-value	HR	95% CI for HR
Univariate analysis			
Sex	0.123	1.908	0.840-4.336
Age at diagnosis, yrs	0.199	1.130	0.985-1.077
Age at IBR treatment, yrs	0.082	1.037	0.995-1.081
n˚ prior therapy lines	0.903	1.023	0.709-1.475
RAI clinical stage	0.485	0.882	0.619-1.255
Leukocyte count, ×10^9^/L	0.484	1.001	0.998-1.005
Hemoglobin level, g/L	0.333	0.991	0.973-1.009
Platelet count, ×10^9^/L	0.901	1.000	0.993-1.006
Deletion 17p	0.782	1.131	0.473-2.704
Deletion 13	0.492	0.739	0.312-1.750
Deletion 11q	0.534	0.739	0.286-1.913
Trisomy 12	0.522	1.615	0.373-7.001
Complex karyotype	0.242	0.510	0.165-1.575
Previous HTA	0.048	2.465	1.009-6.019
Previous CVD	0.028	2.854	1.118-7.284
Multivariate analysis			
Previous CVD	0.031	2.850	1.097-7.169
Age at IBR treatment, yrs	0.092	1.039	0.994-1.085

HR, Hazard ratio; CI, Confidence interval; IBR, Ibrutinib; HTA, hypertension; CVD, cardiovascular disease.

**Table 3:** Univariate and multivariate Cox regression analyses of risk factors for hypertensive toxicity.

Risk factor	P-value	HR	95% CI for HR
Univariate analysis			
Sex, women	0.067	2.934	0.929-9.271
Age at diagnosis, yrs	0.951	0.998	0.942-1.057
Age at IBR treatment, yrs	0.359	0.992	0.975-1.009
n˚ prior therapy lines	0.747	0.915	0.535-1.567
RAI clinical stage	0.453	0.826	0.502-1.360
Leukocyte count, ×10^9^/L	0.830	1.001	0.995-1.006
Hemoglobin level, g/L	0.243	0.985	0.960-1.010
Platelet count, ×10^9^/L	0.957	1.000	0.991-1.009
Deletion 17p	0.666	0.769	0.234-2.533
Deletion 13	0.804	0.854	0.246-2.967
Deletion 11q	0.162	2.365	0.708-7.904
Trisomy 12	0.671	1.565	0.198-12.363
Complex karyotype	0.954	0.959	0.229-4.017
Previous HTA	0.018	6.185	1.362-28.018
Previous CVD	0.024	5.727	1.258-26.077
Multivariate analysis			
Previous hypertension	0.009	7.935	1.677-37.557
Sex, women	0.031	3.463	1.117-10.730

HR, Hazard ratio; CI, Confidence interval; IBR, Ibrutinib; HTA, hypertension.

**Figure 3:**
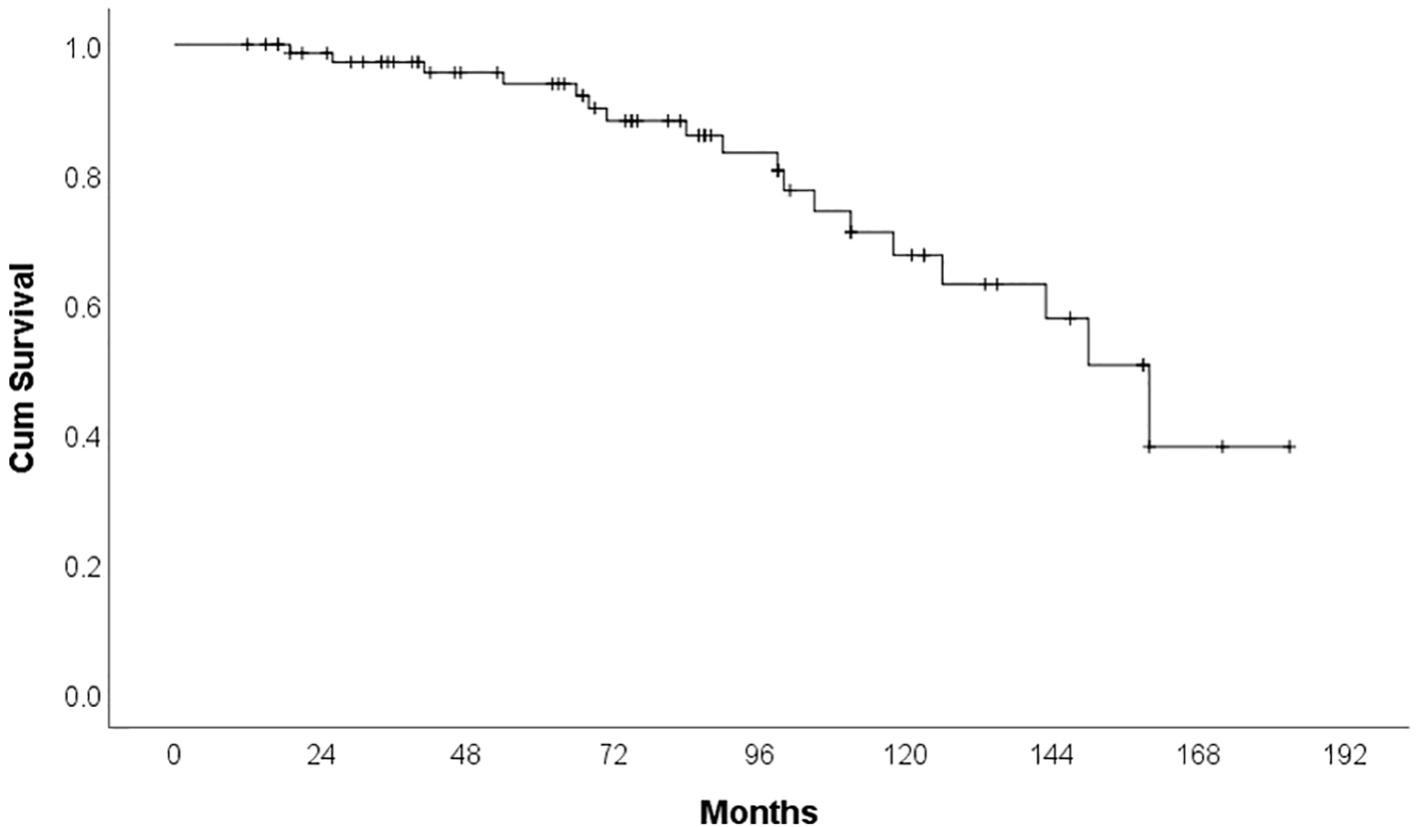
Kaplan Meier curve for overall survival.

By the time of analysis, 18 patients (22.8%) had died. The majority of deaths were attributable to infections (66.6%), followed by disease progression in 27.8%, and sudden cardiac death, which occurred in one patient. The median overall survival was 160 months (95% CI: 137.37–182.63). **Figure 3** shows the overall survival of patients treated with Ibrutinib in our cohort. The development of cardiotoxicity did not significantly impact overall survival (p = 0.714), nor did a personal history of cardiovascular disease prior to Ibrutinib initiation (p = 0.292).

## Discussion

The administration of ibrutinib in patients with CLL has been associated with a more than fourfold increase in the risk of serious cardiac events, including AF, heart failure, and hypertension (12, 13). This study offers insights from a single institution regarding the safety profile of ibrutinib in CLL patients outside the context of clinical trials, with a particular focus on the incidence and clinical implications of cardiovascular toxicity.

Cardiotoxicity was observed in over a quarter of patients (29.1%) during treatment. The most prevalent events included hypertension, AF, and heart failure, often of higher severity grades and frequently necessitating the discontinuation of treatment. Notably, although rare, myocardial infarction and sudden cardiac death underscore the potential for severe cardiovascular outcomes.

Furthermore, ibrutinib was discontinued in 35 patients (44.3%), with 34.3% ceasing therapy due to adverse events. Discontinuation of ibrutinib therapy in CLL patients is more frequently attributed to intolerance rather than disease progression, as corroborated by findings from a multicenter retrospective study involving 616 patients (14). AF and bleeding were the most commonly reported causes of treatment intolerance (15, 16).

Consistent with prior research, hypertension was identified as the most prevalent cardiovascular adverse event in our study, with an incidence rate of 15.2%. A majority of these cases were of greater severity, with 58.3% classified as grade 3, aligning with existing literature (17). A comprehensive meta-analysis of clinical trials indicated that ibrutinib exposure is associated with a 2.82-fold increased risk of developing hypertension (95% CI: 1.52–5.23), including both the exacerbation of pre-existing hypertension and the emergence of new cases (12, 18). Within our study cohort, a personal history of hypertension significantly predicted the occurrence of ibrutinib-related hypertensive cardiotoxicity (p = 0.009; OR = 7.935; 95% CI: 1.677–37.557), with patients facing up to a eightfold increased risk. The significance of this finding is emphasized by the fact that most patients in this context are older adults with a substantial comorbidity burden, with hypertension being the most commonly encountered condition. Furthermore, according to previously published data, there is a potential link between hypertension and sudden cardiac death in patients undergoing ibrutinib-based therapy (19). In our study, one patient experienced sudden cardiac death. Notably, this patient had no history of chronic diseases, including cardiovascular conditions, and was not on any chronic medication. Although the incidence is low, this finding underscores the importance of thorough cardiovascular risk assessment prior to and during ibrutinib therapy, particularly in patients with pre-existing hypertension or other cardiovascular comorbidities.

AF stands out as a prevalent adverse effect, impacting between 3.5% and 16% of patients receiving Ibrutinib therapy (5, 12, 20–25). The exact mechanisms by which BTKi induce AF are not yet fully elucidated, but existing evidence points to a combination of on-target and off-target effects. Specifically, the inhibition of C-terminal Src kinase (CSK) and alterations in the inositol phosphoinositide 3-kinase (PI3K)-Akt signaling pathway, along with possible disruptions in cardiac ion channels, are thought to contribute to the development of arrhythmias and atrial fibrosis (26–30). In our study cohort, 10.1% of patients experienced AF during ibrutinib treatment. The severity of AF was distributed as follows: 25% of cases were grade 1, 37.5% were grade 2, and 37.5% were grade 3. This observation is noteworthy, as AF led to the discontinuation of ibrutinib in 62.5% of the affected patients, highlighting its significant impact on the tolerability of the treatment. Additionally, managing AF often requires the initiation of anticoagulant therapy, which poses a therapeutic challenge due to the increased risk of bleeding associated with the concurrent use of anticoagulants and ibrutinib.

Heart failure has historically been linked to chemotherapeutic agents that inflict direct damage on the myocardium. Recently, however, BTKi, such as ibrutinib, have also become a subject of concern. Although initial clinical trials did not identify heart failure as a significant risk, subsequent analyses of aggregated long-term follow-up data have revealed a delayed yet significant incidence, affecting up to 5% of patients (8, 9). In our study, six patients experienced heart failure while undergoing ibrutinib therapy, with all cases classified as grade 3 or 4 in severity. Importantly, no mild cases (grade 1 or 2) were observed, resulting in the cessation of treatment for all affected individuals. These results highlight the necessity for proactive and frequent cardiac monitoring to detect early, subclinical changes in cardiac function, thereby mitigating the risk of severe complications and maintaining treatment continuity.

Some studies also showed an association between ibrutinib use and the risk of acute myocardial infarction (31). Two acute myocardial infarctions (2.5%) were also recorded in our cohort, both classified as grade 3, characterized by severe symptoms, abnormal cardiac enzymes, hemodynamic stability, and electrocardiogram changes consistent with infarction, according to CTCAE v5.0. These events required the discontinuation of ibrutinib therapy in both cases.

Our study reveals a nearly threefold increase in the risk of cardiotoxicity among patients with a history of cardiovascular disease (HR = 3.463; p = 0.031). This finding emphasizes the necessity of a thorough cardiovascular evaluation before commencing ibrutinib therapy, particularly for those with existing cardiac conditions. Patients with CLL are generally older and often have multiple comorbidities, including cardiovascular disease, which underscores the need for customized monitoring strategies. Detecting subclinical cardiotoxicity early is vital to avert the development of clinical heart failure. Managing AF in patients receiving BTKi involves assessing stroke risk, controlling heart rhythm, and addressing comorbidities such as hypertension and heart failure. Lifestyle adjustments and pharmacological treatments, including blood pressure monitoring and antihypertensive drugs, are crucial. Vigilant monitoring is essential for patients with pre-existing cardiovascular conditions, and any signs of heart failure or significant drug interactions should lead to a cardiologist referral (4). Additionally, prior research has indicated that pre-existing cardiovascular disease correlates with higher rates of atrial arrhythmias and increased mortality in patients with hematologic malignancies treated with ibrutinib, reinforcing the significance of our findings (32).

Furthermore, population-based studies provide valuable context for understanding the overall impact of ibrutinib on cardiovascular health. The cohort study (33) comparing Ibrutinib-treated CLL patients to unexposed controls found a 3-year incidence of 22.7% for AF in ibrutinib-treated patients versus 11.7% in controls, an 8.8% risk of hospitalized bleeding versus 3.1% in controls, and a 7.7% risk of heart failure versus 3.6% in controls. However, that study did not find a significant difference in the risk of ischemic stroke or AMI, adding nuance to the overall cardiovascular risk profile associated with ibrutinib.

In contrast, our study did not identify significant associations between cardiotoxicity and variables such as age at diagnosis, number of prior treatment lines, clinical stage at diagnosis, or common laboratory parameters (e.g., leukocyte count, hemoglobin, and platelet count). However, existing literature on patients treated with BTKi, including ibrutinib and acalabrutinib, has identified certain risk factors for cardiotoxicity. These studies indicate that older patients and those with heart failure with reduced ejection fraction are at an elevated risk for developing atrial fibrillation/flutter. Additionally, risk factors for ventricular arrhythmias, such as ventricular tachycardia/fibrillation and sudden cardiac death, have been identified in patients with male gender, obesity, hypertension, systolic heart failure, and a history of myocardial infarction (34). Furthermore, cytogenetic abnormalities did not correlate with an increased risk of cardiotoxicity in our study cohort. Studies utilizing Next-Generation Sequencing (NGS) have identified specific genotypes, including GATA4 rs804280 AA, KCNQ1 rs163182 GG, and KCNQ1 rs2237895 AA, as being associated with ibrutinib-related cardiovascular side effects. A high genetic risk score, defined by the presence of at least two of these genotypes, was linked to an 11.5-fold increased risk of cardiovascular side effects (p = 0.019; 95% CI, 1.79-119.73), which was not explored in our study (35, 36). Another observation from our study is that the number of prior treatment lines did not significantly contribute to the development of cardiotoxicity. The absence of a significant association between the number of prior treatment lines and the development of cardiotoxicity suggests that cardiovascular risk is not confined to heavily pretreated patients. Consequently, careful cardiac monitoring should be ensured for all patients, including those receiving first-line therapy. Interestingly, while cardiotoxicity often led to the cessation of treatment, it did not show a significant correlation with decreased overall survival. This outcome may be indicative of effective toxicity management and thorough monitoring in clinical settings. Nevertheless, certain studies have reported a stronger link between cardiotoxicity and diminished overall survival in patients undergoing BTKi therapy (29), highlighting the scarcity of real-world data on this issue. These observations imply that although cardiotoxicity can result in treatment discontinuation, it does not necessarily equate to worse survival outcomes in the short to intermediate term.

The limitations of our study include the fact that, although provides real-world evidence, its findings would have been further strengthened if it had been conducted as a multicenter study, allowing for a broader and more representative patient population, thus improving the external validity of the results. Additionally, the absence of a dedicated cardio-oncology team represents a limitation in terms of the consistency of cardiovascular assessments. Cardiac evaluations were performed by various cardiologists across different institutions, which may have introduced variability in diagnostic approaches and interpretation.

## Conclusion

Our study emphasizes the importance of monitoring and managing cardiovascular adverse events in patients treated with ibrutinib for CLL. Patients with pre-existing cardiovascular comorbidities are at particularly high risk and may benefit from individualized treatment planning, closer follow-up, and consideration of alternative BTK inhibitors or bcl-2 inhibitor. Prospective studies are warranted to refine risk stratification tools and to further elucidate the mechanisms underlying BTK inhibitor-associated cardiotoxicity.

## Data Availability

The original contributions presented in the study are included in the article/supplementary material. Further inquiries can be directed to the corresponding authors.
